# Exploring the concepts of decent work through the lens of SDG 8: addressing challenges and inadequacies

**DOI:** 10.3389/fsoc.2023.1266141

**Published:** 2023-11-20

**Authors:** Bianca Ifeoma Chigbu, Fhulu Nekhwevha

**Affiliations:** Department of Sociology and Anthropology, University of Fort Hare, Alice, South Africa

**Keywords:** decent work, sustainable economic growth, SDG 8, gender inequality, market economies, informal sector, environmental sustainability

## Abstract

Promoting decent work and sustainable economic growth within the framework of Sustainable Development Goal 8 (SDG 8) entails addressing gender inequality, the consequences of market economies, and the role of the informal sector while also considering environmental sustainability. Research on SDG 8 remains limited, often adopting an appraisal perspective, and the concept of decent work within this goal remains relatively unexplored. Additionally, the focus on the challenges and inadequacies of achieving sustainable economic growth through decent work in the context of SDG 8 is insufficient, resulting in significant knowledge gaps. To contribute to filling these gaps, this paper adopts a descriptive and critical review perspective, systematically analyzing 108 journal papers and reports to investigate the concept of decent work within SDG 8. The research addresses the challenges and inadequacies related to decent work embedded in SDG 8. The review reveals that while progress has been made in tackling gender inequality in the labor market, gender bias, income discrepancies, and underrepresentation of women in senior positions persist, hindering inclusive and sustainable economic growth, full and productive employment, and decent work for all – SDG 8. Moreover, SDG 8’s focus on inclusive and sustainable development falls short of effectively addressing market economies’ structural disparities, insecure working conditions, and exploitative labor practices. Additionally, support for informal sector workers, who lack essential rights such as legal protection and social security, remains insufficient. Ecological destruction is sometimes an unintended consequence of purely market-based labor markets with an emphasis on economic growth, with SDG 8 lacking sufficient integration of environmental sustainability in its framework. The novelty of this study comes from its in-depth, critical, and policy-focused analysis of the ideas around decent employment in the context of SDG 8. The findings underscore the importance of providing fair, safe, and secure employment opportunities to support economic growth and development while upholding workers’ rights. In conclusion, we emphasize the crucial role of promoting decent work and sustainable growth in achieving SDG 8’s overall objectives, as it directly impacts other SDGs.

## 1 Introduction

The United Nations (UN) has adopted the Sustainable Development Goals (SDGs) to guide efforts toward achieving development that is sustainable by 2030. These goals, known as “transforming our world,” outline an ambitious agenda (Rai et al., 2019). Among these goals, SDG 8 promotes sustainable growth, employment, and decent work for all individuals (Küfeoğlu, 2022). One of the targets of SDG 8 is to achieve “economic growth” through the concept of “Decent Work” across all member nations of the UN (Küfeoğlu, 2022). The notion of work emerged alongside discussions on sustainability in the 1990s and its relationship with economic growth (Frey, 2017). Scholars argue that SDG 8 encompasses workforce diversity opportunities for all individuals, including people with disabilities, gender equality, and fair wages for everyone involved (Khalique et al., 2021). It emphasizes creating a safe working environment that’s equally accessible to both men and women in terms of employment opportunities (United Nations, 2015). The target for ensuring work for all entails providing opportunities and fair compensation that contribute to overall economic development (Khalique et al., 2021).

Research on SDG 8 remains scarce, and the existing papers tend to take an appraisal perspective, while the concept of decent work within SDG 8 remains relatively unexplored. Further, the focus on the challenges and inadequacies of sustainable economic growth through “Decent Work” in the context of SGD 8 seems scanty, leaving significant knowledge gaps. To contribute to filling these knowledge gaps, this paper adopts a descriptive and critical review perspective. We delved into the concept of decent work and examined the challenges and inadequacies in its pursuit within the framework of SDG 8. By analyzing various perspectives and dimensions, this research aims to shed light on the complexities surrounding decent work and contribute to the ongoing discourse on sustainable development. Our analysis shows that despite the international commitment to promoting decent work and economic growth, significant challenges persist in achieving these objectives. Based on our findings, gender inequality continues to pose a considerable barrier to realizing decent work opportunities for all. The results of this review show that the injustices inherent in purely market-based labor markets also raise questions about the compatibility of economic growth with social and environmental sustainability. Additionally, neglected realities surrounding informal work and the need to integrate ecological sustainability further complicate the pursuit of decent work within the framework of SDG 8. These issues highlight the urgent need for a comprehensive understanding of the challenges and inadequacies in pursuing decent work and economic growth.

This study is significant since Pereira et al. (2019) noted that empirical research on decent work is still in its infancy and that most studies find a deficiency in decent work and do not cover the entire idea of decent labor. According to Fontana and Oldekop (2020), this is a necessary time to reflect on the SDGs. Efforts to implement initiatives connected to the SDGs have increased, but we are still in an early stage when adjustments and reorientations are feasible. Thus, now is the perfect opportunity to present ideas and facts that could inform ongoing discussions on the SDGs and their implementation (Fontana and Oldekop, 2020). Our research is a guiding light in the academic community, revealing critical aspects of decent employment under SDG 8 and, by extension, sustainable economic growth. In the continued search for a more egalitarian, inclusive, and sustainable global economic environment, its extensive analysis, critical insights, and policy relevance made it not just significant but essential. Furthermore, as global efforts to implement initiatives connected to the SDGs continue, this research offers timely and relevant information to inform ongoing discussions and adjustments.

Through this study, we contribute to the existing body of knowledge in several ways. Firstly, we critically examine the relationship between gender inequality and decent work within the context of SDG 8. By highlighting the impact of gender disparities on employment opportunities and the overall well-being of individuals and societies, this research aims to inform policy and practice for creating more equitable work environments. Secondly, this study delves into the injustices of market economies and explores their contribution to decent work. By questioning the prevailing economic system and its impact on social and environmental dimensions, this research offers insights into potential strategies for rethinking the pursuit of economic growth and decent work. Thirdly, the study addresses the challenges and neglected realities of informal employment and the economy within SDG 8. By examining the vulnerabilities and opportunities associated with informal work, we aim to inform policies that protect and empower informal workers, fostering inclusive and sustainable economic growth. Lastly, this research underscores the need to strengthen the integration of environmental sustainability within the framework of SDG 8. By analyzing the ecological dimensions of decent work, it advocates for a holistic approach that considers the long-term environmental impacts of economic activities. Our research objectives are as follows:

- To examine gender income disparity and the achievement of decent work within the context of SDG 8.
- To explore the injustices inherent in market economic systems, examining their numerous contributions to the terrain of decent labor.
- To shed attention on the complicated difficulties contained within informal employment and the larger economy, which are sometimes disregarded within the scope of SDG 8.
- To emphasize the critical need to strengthen the integration of environmental sustainability into the fundamental fabric of SDG 8.

The study starts by elucidating the data type and collecting methods used to fulfill the purpose of the investigation. The second part examines the study results and organizes them into pertinent topics relating to obstacles and shortcomings in the pursuit of decent employment and economic development in accordance with SDG 8. The discussion and conclusion thoroughly explain the intricacies of decent work and its role in attaining sustainable economic growth via a conclusion of the key results and a discussion of the study’s consequences.

## 2 Conceptual framework: decent work within the framework of SDG 8

In 2019, the ILO outlined the concept of decent work, which spans a complex spectrum defined by equal wages, employment security, safe working conditions, and the protection of social welfare measures. At the core of our conceptual framework is the complex intersection between gender inequality and the need for decent employment. Persistent gender prejudices, financial disparities, and the underrepresentation of women in top management positions (Khairy and Ghoneim, 2023) obstruct the achievement of SDG 8 in a glaring manner SDG 8. Complementing this component, we delve into the myriad complexity inherent to market economic systems (Xu and Chakraborty, 2021), which repeatedly generate structural disparities, insecure work circumstances, and the propagation of exploitative employment practices (Chowdhury and Żuk, 2018; Menéndez-Espina et al., 2020; Ranaldi and Milanović, 2022). Our scientific quest delves deeply into the complicated mechanisms that characterize market-driven economies, providing insight into their influence on employment quality, labor rights protection, and the long-term viability of economic ventures. In addition to our research, we examine the complex terrain of the informal sector, a domain characterized by a conspicuous lack of legal protections and social security guarantees, a solid barrier to the acquisition of decent work (Dewick et al., 2022; Padmavathi and Aruna, 2022; Singh et al., 2023). Within this dimension, we examine a repertory of policies and strategic interventions designed to empower the informal labor force and provide them with the resources to improve their economic well-being. We go beyond standard economic paradigms to consider the ecological repercussions of the unrelenting quest for economic development under purely market-based labor market frameworks (Sun et al., 2016; Adebayo et al., 2021).

The unified themes of gender inequality, the necessities of the market economy, the crucial role of the informal sector, and the inescapable requirement of environmental sustainability are unified within our complete framework. The dynamics of this framework are intrinsically interdependent, with one aspect resonating with and enhancing the others. It is observed that gender inequities penetrate economic institutions, impacting both the official and informal labor sectors. Similarly, the market economy’s imprint reverberates throughout the work spectrum, influencing the quality of labor in both formal and informal circumstances. Environmental sustainability is concurrently linked with popular economic models, potentially impacting policy paradigms across industries. This detailed exegesis aims to explain the many complexities underlying decent employment within the scope of SDG 8. The meticulously crafted conceptual framework serves as the compass for our academic investigation, imparting a methodological rigor that enables us to dissect with precision the multifaceted challenges and inherent deficiencies inherent to the aspiration for decent work within the larger context of sustainable development.

## 3 Materials and methods

We conducted a systematic literature review (SLR) to explore the concepts of decent work through the lens of SDG 8 and to address the challenges and inadequacies of decent work embedded in SDG 8. As García-Peñalvo (2022) highlighted, SLR is a way to sift through the vast amount of available digital scientific output to find, evaluate, and make sense of the work of researchers and practitioners on a specific topic. Our foci are grounded in a methodology that provides a defined set of methods and synthesis components drawn from shared databases of high-quality, previously conducted research, arguments, and analyses (Chigbu et al., 2023). Utilizing the SLR technique allowed for a comprehensive, organized, and objective investigation of the current literature on decent employment within SDG 8. It enabled us to identify the sector’s obstacles, voids, and possibilities, providing valuable insights for academics and policymakers. Our SLR process comprised four key stages: preparing research questions, literature search, critical evaluation, developing a logical structure, and data analysis for analysis.

### 3.1 Preparing research questions

We initiated the SLR by creating precise research questions to delve thoroughly into decent work’s complexities in SDG 8. These questions were carefully prepared to drive our search and research, assuring a concentrated examination of gender inequality, the market economy’s implications, the informal sector’s role, and the environmental sustainability of SDG 8’s goal of decent employment.

### 3.2 Literature search

We searched the Scopus database, grey publications from Google Scholar, and simple Google. In addition, we examined UN website publications to improve our dataset. To further our analyses and arguments, the search utilized particular terms and phrases such as “SDG 8 and decent work,” “SDG 8 and limitations,” and “economic growth and decent job.” The search was not restricted by publication date, enabling a full coverage of the field’s current literature.

### 3.3 Conducting critical evaluation

**Figure 1:**
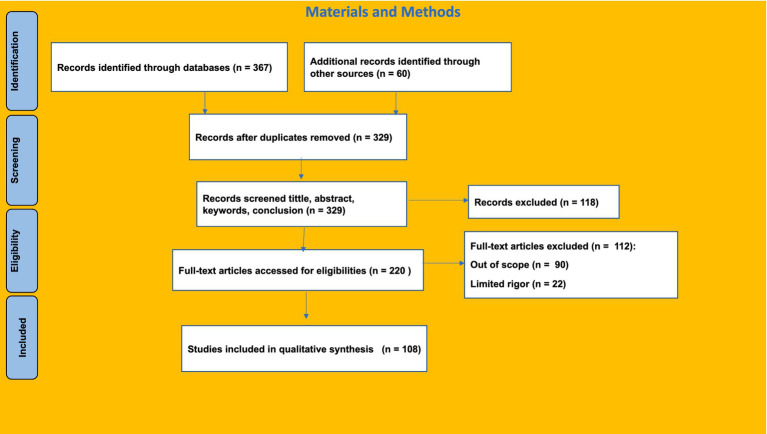
Flowchart illustrating the selection procedure of the systematic literature review.

After considering the publication titles, the keywords and abstracts of the suggested publications were examined. Several papers’ introductions and scope were assessed to determine their relevance. Then, each publication was assessed to determine its relevance to the study. Several works connected to references beyond the scope of the first search were subsequently evaluated utilizing an internet search. Figure 1 illustrates the publication selection process. We thoroughly examined and analyzed each publication’s title, abstract, and keywords in relation to SDG 8 and decent work after obtaining 329 pieces. A first screening decreased the number of publications to 220. Subsequently, a thorough evaluation was conducted, eliminating 90 irrelevant papers and 22 studies lacking sufficient rigor. This investigation led to the final selection of 108 publications that served as the foundation for our research.

### 3.4 Developing a logical structure

In the context of decent employment, we conducted an in-depth study of the selected publications. We discovered recurring themes and patterns concerning gender inequality, purely market-based labor markets, the informal sector, and environmental sustainability. Through thorough classification, these topics were integrated into a logical structure that fully understood the obstacles and deficiencies of attaining decent work and sustainable economic growth under SDG 8.

### 3.5 Data analysis

Using thematic and content analysis, this study investigated the complexity of decent work within the context of SDG 8. Thematic analysis was used to find reoccurring themes and patterns in the literature. Publications were analyzed to extract underlying themes about gender inequality, the market economy’s ramifications, the role of the informal sector, and environmental sustainability in the context of decent work. Content analysis was used to explore the selected publications’ textual content more deeply. We used this strategy to assess textual material to extract helpful information systematically. Thematic and content analysis conclusions were based on a process that included a rigorous investigation of credible existing research, logical reasoning, and appraisals of distinct opinions from the literature reviewed for this study. By integrating content and thematic analysis, we provide a comprehensive and nuanced understanding of the literature in this SLR study.

### 3.6 Limitations of the study

We must recognize the limits of our study. The research relied mainly on available literature, which may contain inherent biases or coverage limitations. In addition, despite our efforts to guarantee a thorough selection procedure, the ever-changing nature of study topics and terminology may have led to an overlook in our search method. Despite these limits, the study gives valuable insights into the difficulties and shortcomings of combining decent employment and economic growth, providing a solid platform for future research and policy development in this crucial field.

## 4 Findings: challenges and inadequacies in pursuing decent work and economic growth

SDG8’s emphasis on decent work and economic development is insufficient; productive employment and decent work for all men and women by 2030 must account for the value and costs of social reproduction since there is no following change in the gender composition of social reproductive activity (Rai et al., 2019). Tensions between productivity-enhancing and decent labor logic hinder the creation of treatments with persistent performance benefits (Hasle and Vang, 2021). The indication is that the focus of SDG 8 remains on GDP and *per capita* growth (Rai et al., 2019), which is problematic (Meurs et al., 2019; Rai et al., 2019; Eberth et al., 2023). For instance, the GDP productive boundary excludes much social reproductive work (Rai et al., 2019). Reproductive activities were not accounted for in the writings of Karl Marx; capitalists exploit workers because they must work more than is required for their reproduction; the excess effort is known as creating surplus value (See: Marx, 1972, 1981). As Frey (2017) puts it, despite the prominence of full employment and decent work in SDG 8, the 2030 Agenda supports market-centered institutional frameworks that may provide difficulties in fulfilling the target. Specifically, the grafting of human rights to full employment and decent work onto a business-oriented economic growth agenda in SDG 8 raises questions about whether the 2030 Agenda recognizes full employment and decent work as state human rights obligations or economic growth benefits (Frey, 2017).

Evidence suggests that the achievement of the rights to full employment and decent labor does not always follow economic development; in fact, studies indicate that, at least in certain circumstances, it is more likely that the reverse causal link prevails (Frey and MacNaughton, 2016). For instance, sociologists like Hammer et al. (2022) emphasize that significant formal job increases have not been made despite industrial progress. In reality, the informal sector predominates and is characterized almost universally by subsistence pay, employment, and social instability, all of which fall under immoral work (Hammer et al., 2022). The problem is that the working conditions of those employed in the unorganized sector are risky, lack social protection, and are polluting, which increases the likelihood that employees will get sick or injured and jeopardize their health and well-being (Adei et al., 2021).

According to an ILO assessment from 2019, most countries still have a long way to go before attaining inclusive and decent employment for all, and development on SDG 8 was slowing down. According to Hammer and Ness (2021), from a socio-political perspective, the agenda of SDG 8 and similar interventions fail because not enough attention is given to the heterogeneity and complexity of intersecting social and material relations that support informal and precarious work and its role in capital accumulation. For instance, businesses intentionally alter the makeup of their workforce to limit labor’s ability to negotiate and lower the value of labor power (Chigbu and Nekhwevha, 2022c). The issue is that unionized workers are more likely than their non-union colleagues to speak up against indecent workplaces, which includes an increasing preference for non-unionized female workers and rural and semi-rural workers (Hammer and Ness, 2021; Sojourner and Yang, 2022). Critical factors influencing capital accumulation with consequences for the structure and differentiation of the workforce include the asymmetrical power relations between capital and labor and the state’s involvement in institutionalizing a flexible labor regime (Hammer and Ness, 2021).

Having given the introduction to this section, our research objectives are methodically constructed and accomplished to grapple with the complexity of creating decent employment within the auspices of SDG 8. First, we thoroughly examine the delicate link between gender income disparity and the achievement of decent work within the context of SDG 8. Second, our scientific investigation extensively explores the injustices inherent in purely market-based labor markets, examining their numerous contributions to the terrain of decent labor. The third goal of the study is to shed attention on the complicated difficulties contained within informal employment and the larger economy, which are sometimes disregarded within the scope of SDG 8. Finally, our research emphasizes the critical need to strengthen the integration of environmental sustainability into the fundamental fabric of SDG 8. These problems illustrate the vital need for an in-depth analysis of the challenges and shortcomings of pursuing decent employment and economic expansion.

### 4.1 Gender and income inequality: decent work

*Gender disparities in the labor market remain a persistent challenge and should be at the heart of decent work discourse because women often face barriers to accessing decent work, including gender-based discrimination, wage gaps, and limited representation in decision-making positions. Achieving gender equality in the workplace requires addressing these challenges and promoting policies that ensure equal opportunities, fair pay, and work-life balance for all genders.* Gender equality is essential for both sustainable development and economic prosperity. Keeping in mind the SDG 8 goals, the objective is to achieve full and productive employment and decent work for all women and men by 2030. The problem is that when women enter the workforce, they are less likely than males to find employment (ILO, 2017). For instance, Statistics South Africa (2021) shows that the unemployment rate for women in South Africa is 8.1% points greater than that of males. Men are more likely than women to have paid employment regardless of race. Still, women are more likely than males to be engaged in unpaid work, even in Asia and Germany, indicating that the labor market is more favorable to men than women (ILO, 2020; Toczek et al., 2021).

Many variables, such as long-standing institutional impediments, socioeconomic and technical development, and economic drive and shocks, persistently drive gender inequalities in the workforce (WEF, 2022). Because of the pervasive gender disparity in the labor market (Khairy and Ghoneim, 2023), women have fewer opportunities to advance professionally and raise their salaries than men (Khairy and Ghoneim, 2023). This is especially true in male-dominated fields such as engineering, construction, and information technology (Khairy and Ghoneim, 2023). Among the factors that make it difficult for women to get acceptable employment are the “glass ceiling” and other forms of workplace discrimination against female employees, working conditions, and occupational segregation, all of which are significant challenges to overcome (de Pryck and Termine, 2014; Khairy and Ghoneim, 2023). According to the International Labor Organization’s 2017 World Employment and Societal Outlook – Trends for Women, social norms and a variety of socioeconomic obstacles, including work-life balance, marital status, and lack of transportation, are preventing women from advancing in the workforce (de Pryck and Termine, 2014). Also, economic hardships, sexism, and racism experiences have historically prevented women from accessing decent employment (Autin et al., 2022) and have been significant across industries (Fabry et al., 2022).

According to Ferrant et al. (2014), women spend 2–10 times as much time as males on unpaid care labor. The uneven allocation of caring obligations is connected to discriminatory social structures and stereotypical views on the appropriate roles for men and women. The examination of gender disparities in labor outcomes, such as labor force participation, salaries, and job quality, fails to take into account gender disparity in unpaid care work (Ferrant et al., 2014), and this is one of the missing links. As Vyas (2021) put forward, it is no longer acceptable to attribute women’s stagnation in the labor market to a lack of education or concerns about their suitability for a particular position. Despite their credentials, women all over the globe are taking on the dual duty of caring for others and working, and as a result, they often have to accept insecure jobs. This is because women must perform unpaid care labor due to family commitments (Vyas, 2021). The position of women in the labor market and their status and authority inside the family are all impacted by the male–female wage gap (Witkowska, 2013; Toczek et al., 2021).

In conclusion, according to the literature, objective 8.5, which calls for full and productive employment, decent work for people of both genders and equal remuneration for labor of equal worth, has not yet been averagely attained. Objective 8.8 goal of promoting safe and secure working conditions for all employees, particularly those in precarious employment (women), is currently difficult to achieve. SDG 8 seeks to promote decent jobs and economic prosperity for all persons, but continuing gender inequities in the labor market pose substantial hurdles to attaining these goals. Gender discrimination, income inequalities, and a lack of representation in leadership roles are just a few of how women’s careers are held back (Menéndez-Espina et al., 2020; Smith et al., 2021). To promote gender equality in the workplace, sustainable development, and economic success, these obstacles must be overcome. Women already experience discrimination in the workplace because of their gender, and the salary difference, poor working conditions, and occupational segregation only worsen things. Again, women’s access to safe, respectable jobs is hampered by the time and energy required for unpaid care duties.

Policymakers and stakeholders must apply various strategies to overcome these problems and realize SDG 8’s goals and targets 8.5 and 8.8:

- Promoting Equal Opportunities: Enacting rules that eliminate discriminatory practices and obstacles to access in the workplace for both men and women.
- Supporting Work-Life Balance: Policies promoting work-life balance should be enacted to aid working mothers in juggling their professional and personal lives.
- Closing the Wage Gap: Equal pay for equal effort, as well as addressing the gender wage gap via equitable compensation methods, is essential to closing the wage gap.
- Reversing Gender Roles in the Workplace: Promoting female engagement in historically male-dominated fields via strategic programs and initiatives.
- Creating Safe and Secure Work Environments: Prioritizing the promotion of safe and secure working conditions, especially for women in precarious employment, is crucial to creating safe and secure work environments.
- Addressing Unpaid Care Work: Taking up the issue of uncompensated care work means advocating for an equal allocation of caregiving obligations and recognizing and respecting uncompensated care labor.
- Challenging Gender Norms: Promoting gender equality through combating the assumptions and discriminatory practices that keep women at a disadvantage in the workplace is a vital part of the movement to challenge gender norms.
- Investing in Education and Skills Development: Investing in education and training helps women acquire the knowledge and abilities they need to secure stable employment.
- Advocating for Women’s Empowerment: Pushing for more opportunities for women to take on positions of influence in organizations and society.
- Data Collection and Monitoring: Strengthening data collecting and monitoring mechanisms to measure progress toward SDG 8 and eliminating gender inequalities in the workplace.

Prioritizing and executing these policy measures may help nations make substantial progress toward achieving SDG 8 and other associated SDGs by reducing the gender gap in the labor market and fostering decent employment for everyone. The labor market and inclusive and sustainable economic development are bolstered when barriers to gender equality and suitable employment are removed.

### 4.2 The injustices of market economy: rethinking sustainable development goal 8

The struggles for decent work and economic growth within the purely market-based labor markets have been multifaceted and debated for many years. While market economies have promoted innovation, efficiency, and overall economic growth, they also face challenges that can hinder decent work opportunities, equitable economic progress, and SDG 8. The rise of capitalism was the catalyst for both current economic expansion and the fight for decent employment. The quest for purely market-based labor markets to compete globally has led to economic concentration (Sell, 2020). Given that capitalism is inherently growth-oriented and is based on the logic of competition and accumulation (Hall and Davis, 2021), it produces the modern problems of unemployment and labor-capital conflicts (Xu and Chakraborty, 2021), as well as income inequality (Chowdhury and Żuk, 2018; Ranaldi and Milanović, 2022).

The emergence of purely market-based labor markets resulted in a significant reduction in human well-being across all areas, with salaries falling below the minimum necessary for sustenance and a decline in human size (Sullivan and Hickel, 2023). Like never before in the history of capitalism, the global economic geography has undergone a significant transformation in terms of the magnitude and content of international flows of commodities, services, and financial assets, as well as the localization of industrial activity, mainly manufacturing (Ricci, 2021). The liberalization of local and international markets for commodities, capital, and labor, pushed via a wide range of neoliberal economic policies by almost all governments worldwide, has been the defining feature of this period (Ricci, 2021). Global value chains have been claimed to lead to new types of worker poverty, and many sectors’ mostly female workforces are hyper-exploited (given salaries below the level necessary for subsistence) (Selwyn, 2019). Workers in a market economy are under tremendous pressure to meet very high and rising productivity goals, receive base pay that is insufficient to cover their needs for individual and social reproduction, put in a lot of overtime, and as a result of these combined pressures, suffer physical and emotional degradation (Selwyn, 2019).

Yang et al. (2021) emphasized that capitalists are engaged in “wage theft,” which is the illegal underpayment or non-payment of employees’ wages and that even workers’ minimal legal rights are not enforced (Cole et al., 2022). This also includes paying less than the minimum wage, refusing to pay for labor during regular business hours or authorized overtime, denying breaks, and making employees pay for supplies or damage (Bernhardt et al., 2009). As work has become more precarious, marked by uncertainty, low income, and limited social benefits and entitlements (Menéndez-Espina et al., 2020), it is regrettable that even the advanced economy with a well-established legal and institutional framework has failed to enforce even basic legal standards (Sell, 2020; Yang et al., 2021). The necessity for capital to extract surplus value from labor leads to wage theft, and most obviously, unlawful underpayment allows management to enhance actual surplus value by documenting legal salaries while reducing cash payments (Yang et al., 2021). Despite these alarming issues, workers participate in their exploitation and work in precarious conditions due to inadequate work protection, which may have severe consequences for productive employment and decent jobs, − which are essential components of Goal 8 of the SDGs (Silaban et al., 2021). Additionally, encouraging precarious positions may make it more challenging to implement Goal 8 on decent work (Silaban et al., 2021).

Regardless of a country’s degree of development, critical personnel are often underpaid by 29%, with this percentage being notably high in high-income nations that employ a significant proportion of foreign workers (ILO, 2023). Many of these employees in several essential industries have little unionization and collective bargaining coverage or none at all (ILO, 2023). These problems are seen as a sort of contemporary slavery known as labor exploitation, in which a person is made to work against their will, is under their boss’s authority, and is compelled by economic circumstances to accept inadequate compensation (Yang et al., 2021). Despite the substantial economic expansion, there is constant proof of worker exploitation in the periphery of the new global economy and labor pressure in older, more established industry centers (Mayer and Pickles, 2010).

Through innovation and market competition, purely market-based labor markets offer opportunities for skills development and adaptation; however, due to its emphasis on profit-driven market dynamics, rapid technological advancements, and the allocation of resources based on market forces, market economies have impacted skills mismatch in the labor market. Due to this, there is a discrepancy between the talents that companies value and those that job seekers possess (Livingstone, 2019; Chigbu and Nekhwevha, 2022a; Chigbu et al., 2023). Due to the capacity of market economies to develop and adapt new technology, the labor market is constantly disrupted and altered (Min et al., 2019; Minbaeva, 2021; Lefèvre et al., 2022). Additionally, the emphasis on efficiency and competitiveness under capitalism pressures companies to change and continue to be successful. This need for efficiency often leads to the automation of repetitive operations and the need for higher-level abilities that can complement and collaborate with technology (Brugger and Gehrke, 2018; Ra et al., 2019; Chigbu and Nekhwevha, 2022b). The consequence might be a skills mismatch as people lacking the required abilities may struggle to find acceptable jobs. Variability and unpredictability, upskilling, deskilling, and skills polarization (Mishra et al., 2019), the replacement of people with machines in the workplace, and the “deskilling” of existing functional jobs (Braverman, 1998) are all contributing to the unskilled proletariat.

The SDGs’ sustainability goals are violated by Goal 8 (Hickel, 2019: 1) because the “global growth of 3% per year renders it empirically infeasible to achieve (a) any reductions in aggregate global resource use and (b) reductions in CO2 emissions rapid enough to stay within the carbon budget for 2°C.” While SDG 8 is based on the idea of enhancing the relationship between a business and its workers, job automation has the opposite impact, undermining the fundamental notion of decent employment and leading firms away from rather than toward the fulfillment of SDG 8 (Braganza et al., 2021). These are traits of degrading employment practices and market economies’ labor restructuring. Many struggle to find quality employment because they lack the skills to prosper in the changing industry. To foster economic development and provide fair employment opportunities, it is critical to close the skills gap via education, training, and lifelong learning (Chigbu et al., 2023).

As production increases, the workforce does not experience the same positive growth in the manufacturing industry (Chigbu and Nekhwevha, 2022c). This might be the reason why critics have emphasized that expanding productivity as stipulated in SDG 8 has not resulted in a concomitant improvement in the standard of living of people, and sustainable development cannot be based on further economic growth (Robra and Heikkurinen, 2019). The inequities inherent to the systematic processes of exploitation that are distinctive of market economies are not considered by SDG 8 or its larger sustainability agenda (Bianchi and de Man, 2021). The logic of growth, competition, and profit-making that underpin the continuous development and expansion of specific industries are in opposition to the SDG-led agenda (Bianchi and de Man, 2021). The idea of sustained and inclusive growth reinforces the primacy of capital and market notions of justice and continues to perpetuate a growth-driven development model rather than addressing the structural injustices that entrench inequalities and reproduce exploitative labor practices (Bianchi and de Man, 2021). The SDGs are yet another international instrument, as stated by Milivojevic et al. (2020), that makes rhetorically solid commitments to the intersections of labor, migration, and exploitation but lacks the clarity and operational strength it requires to lead the path in reducing, if not eliminating, such exploitative practices.

The elements that support or contribute to the circumstances for labor exploitation are still not taken into account by this tool (Milivojevic et al., 2020). Trebilcock (2005) linked a lack of competent governance to a lack of decent work. A lack of governance, poor institutions, and contradictory political commitments may hinder progress on human rights concerns such as target 8.8’s labor rights.^1^ The commitment of target 8.6 to dramatically improve the labor market situation of young people of NEET by 2020 was not met; for instance, see statistics in studies by Cieslik et al. (2022) and Picatoste and Rodriguez-Crespo (2020). Political will, commitment, and the structures and mechanisms for proper governance are essential (Trebilcock, 2005) in achieving decent work within SDG 8. Sadly, the new 2030 development plan favors market-based economic growth tactics above the attainment of everyone’s entitlement to full employment and a reasonable standard of living (Frey and MacNaughton, 2016). In this scenario, SDG 8’s expression and measurement of economic progress continue to leave many people behind (Meurs et al., 2019) due to the ongoing rise in insecure employment (Silaban et al., 2021) and increasing worker exploitation (Selwyn, 2019).

In conclusion, of this section, the advent of market economies has been connected with contemporary economic expansion and the fight for decent labor. This complex link suggests that the objective of a decent job cannot be wholly realized within the existing economic system. Purely market-based labor markets, driven by competition and accumulation, have spawned various problems, such as unemployment, labor-capital disputes, and charges of exploitation and underpayment. Moreover, the growth of market economies has resulted in a decline in human well-being, including subsistence-level income and detrimental effects on human height. The impact of capitalism has also transformed the global economic geography, spurred by neoliberal policies that have liberalized markets and led to new types of worker poverty, which disproportionately afflict female workers. The ubiquitous problem of “wage theft,” which involves illegal non-payment or underpayment of workers’ wages, occurs even though the most fundamental legal norms are not enforced under insecure working circumstances. Despite SDG 8’s focus on inclusive and sustainable development, capitalism’s structural inequities and exploitative labor practices are not sufficiently addressed. In addition, the SDGs’ pledges to address labor exploitation and the interconnections of labor and migration have not been translated into adequate operational capacity to minimize or eradicate exploitative behaviors. With unstable labor on the rise, securing suitable employment remains a challenge. Economic circumstances continue to promote labor exploitation, defined by forced work and insufficient remuneration, as contemporary slavery. Extreme income and wealth disparities must be addressed for human rights, health, political and economic stability, and world peace. It is vital to challenge market economies’ prevailing growth paradigm and promote a counter-hegemonic discourse to address these challenges and those linked with informal labor successfully.

### 4.3 Challenges and neglected realities of informal work and economy within SDG 8

The SDG8 may not adequately address workers’ challenges (home-based workers, street vendors, domestic workers, waste pickers) in the informal sector, who often lack legal protection, social security, and essential benefits such as healthcare and pensions. Overcoming the challenges associated with the informal economy is vital to achieving decent work and inclusive economic growth. According to Tucker and Anantharaman (2020), “informal work” refers to various income-generating activities not covered by state labor laws and wage relations. In addition, “informal sector employers are frequently harsh, ignore the law, and easily dismiss their workers with little to no recourse to legal remedies” (Bonner and Spooner, 2011). For Dewick et al. (2022), the nature of informal work is heavily polluted with illegal activities such as disguised child labor (Singh et al., 2023), which goes unmonitored by any form of government (Padmavathi and Aruna, 2022). It has been argued that the market and state have failed in delivering optimum economic welfare to informal workers even in the case of well-planned and highly urbanized cities (Singh et al., 2023), as it is sometimes stigmatized as troublesome and unmanageable (Padmavathi and Aruna, 2022). Even while the informal economy contributes to the GDP (Charmes, 2012; Dell’Anno and Adu, 2020) and overall production and consumption in both developing and developed countries (Horodnic et al., 2022), it is sometimes not included in a nation’s GDP, unlike the formal sector (Padmavathi and Aruna, 2022). In a different view, women often engage in informal work, particularly in domestic services, which is undervalued and unrecognized, perpetuating negative stereotypes (Jackson, 2019). Critics have expressed worry about the escalating disparities surrounding the gender wage gap, particularly about the unpaid labor that women engaged in domestic work in the informal sector (Jackson, 2019; Singh et al., 2023). Addressing this issue involves recognizing and valuing unpaid domestic work, challenging negative perceptions, and ensuring equal opportunities for all genders (Jackson, 2019). To promote sustained inclusive and economic growth for all in line with SDG 8, it is crucial to prioritize gender equality (Jackson, 2019).

Unfortunately, trade unions and other workers’ organizations undoubtedly face many real challenges in organizing the informal workforce, irrespective of sector or country (Bonner and Spooner, 2011), due to a lack of tradition of democratic functioning, financial self-reliant to pay regular membership dues, legal and regulatory framework, stable and specific workplaces, worker identity and undefined workplaces (Bonner and Spooner, 2011). The exclusionary aspect of organized trade unions inadvertently excludes a sizable segment of the workforce. The vulnerability of informal workers is exacerbated by their lack of legal recognition (Routh, 2015; Bernal-Torres et al., 2019), which contradicts the fundamental idea of decent work enshrined in SDG 8. Furthermore, SDG 8 underlines the significance of providing safe and secure working conditions for all workers, regardless of employment status. However, the lack of legal protections exposes informal workers to exploitative behaviors, undermining the promotion of fair working conditions (Williams and Youssef, 2014). Furthermore, the absence of knowledge and awareness about rights and the existence of trade unions among informal workers impedes the achievement of SDG 8. The SDGs view decent work as requiring workers to be empowered with knowledge and participate in decision-making processes, hampered by this lack of awareness.

Too often, policy elites, including those promoting sustainable development, overlook that informal workers produce urban economies with social and environmental value (Tucker and Anantharaman, 2020) and promote the circular economy and sustainable development (Di Maio and Rem, 2015). The industry is crucial to survival and, more likely, continued livelihoods for persons with low skill levels (Jackson, 2019). However, growth-oriented economies – the core of SDG 8 – reproduce environmental deterioration, economic inequality, and poverty, the exact factors that drive so many people to engage in informal labor (Tucker and Anantharaman, 2020). The fundamental problem is that policymakers still do not have a reliable KPI for boosting the informal sector (Di Maio and Rem, 2015). Despite efforts to integrate the Decent Work agenda into social policies and programs, political processes are structurally unstable; therefore, there are no precise strategic approaches for addressing a capacity gap particular to the informal sector (Dhakal and Burgess, 2021).

Our primary conclusion in this section is that SDG 8 may not sufficiently address the issues faced by workers in the informal sector, resulting in a lack of legal protection, social security, and decent working conditions. Casual workers, who constitute a substantial component of the labor population in several developing nations, engage in income-generating activities outside of official labor regulations and are vulnerable to exploitation by employers who often flout the law. Consequently, the government does not oversee several aspects of indecent labor in this area. In addition, the gender disparity gap, especially in terms of unpaid labor undertaken by women in domestic employment, is a worrying problem that reinforces negative stereotypes and necessitates equitable opportunity and acknowledgment of unpaid domestic work. Unfortunately, authorities often disregard the substantial contribution of informal labor to urban economies, social value, and environmental sustainability. In addition, unions and workers’ groups confront obstacles in organizing the informal workforce, such as the absence of a history of democratic functioning, financial independence, legal and regulatory frameworks, stable workplaces, worker identity, and unclear work arrangements. For fostering sustainable development, reaching SDG 8, and contributing to reducing carbon emissions that harm the environment, it is crucial to recognize the informal sector’s significance and establish appropriate metrics to support its growth.

### 4.4 The need to strengthen the integration of environmental sustainability within the framework of SDG 8

The phenomenon of indecent workplaces poses existential severe threats to achieving SDG 8 (Raimi, 2020). For example, due to growing unemployment rates and greater levels of precarious employment in the labor market, Sub-Saharan Africa has a low inclination toward decent work (Raimi, 2020), while their focus is on economic growth that might hamper the environment. Scholars have argued that SDG 8 is presently part of a climate delay argument since the inclusion of economic development and the emphasis on boosting employment both contribute to unsustainable and unfair results (Kreinin and Aigner, 2022), severe several environmental and human health problems (Sun et al., 2016). For example, China is the world’s manufacturing powerhouse, with manufacturing accounting for approximately 30% of total economic production. The fast economic growth of China and the expanded availability of goods and services raise the need for energy, such as hydroelectricity usage. Sure, hydroelectricity consumption hurts CO2 emissions, which helps to mitigate environmental degradation; however, it also has adverse environmental and societal risks, such as degenerated wildlife habitat, worsened water quality, obstructed fish mobility, and shrunk recreational opportunities on rivers, which cannot be overlooked (Adebayo et al., 2021). China’s economic development, for example, has dramatically decreased poverty in recent decades, allowing it to enter the ranks of upper-middle-income nations. Unfortunately, its economic prosperity has been accompanied by substantial decreases in air quality; in fact, its air pollution has increased far quicker than its economic development (Dang and Serajuddin, 2020). Another troubling problem is that as many as 96 nations lack data on SDG 13, “Climate Action,” making it difficult to adequately assess progress across countries and time (Dang and Serajuddin, 2020).

There has been much discussion on absolute decoupling, which happens when environmental problems are decreased but economic development is maintained. Since capitalism, no matter where it is, is prone to crises, growth-dependent, and market expansion, it is impossible, according to the present state of climate science, for there to be a time of no growth or “de”-growth under market economies (Hall and Davis, 2021). However, although relative decoupling has been demonstrated in certain nations, total decoupling remains challenging owing to purely market-based labor market growth and dynamics (Ward et al., 2016). GDP growth would have to be significantly decoupled from energy and material usage and environmental repercussions for it to be sustainable; yet, there is no evidence that GDP growth can be detached in the long run (Ward et al., 2016). Nevertheless, at the core of green manufacturing is the need for the industry to combine numerous goals, including equitable development, environmental protection, and productivity, via a plethora of regulatory frameworks (Fu and Irfan, 2022).

In conclusion, while SDG 8 target 4 aims to “improve progressively, through 2030, global resource efficiency in consumption and production and strive to decouple economic growth from environmental degradation,” it does not explicitly emphasize the need for sustainable economic practices that minimize environmental impacts caused by intense economic competition and growth-oriented market economies. In a neoliberal capitalist society, pursuing GDP development often comes at the price of ecological sustainability. In addition, China’s industrial supremacy and economic growth have resulted in a substantial rise in energy use, notably hydroelectricity, which has had detrimental environmental effects. The difficulty is achieving complete decoupling, in which economic expansion continues but environmental consequences decline. This work becomes more complex, particularly given that many nations lack adequate data on climate action, highlighting an urgent need for enhanced monitoring and reporting methods.

## 5 Discussion – complex path to SDG 8: unraveling challenges and charting future courses

The global community faces numerous hurdles in achieving SDG 8. This debate goes into four crucial study findings, each of which sheds light on critical impediments impeding the achievement of SDG 8’s objectives. We explore the intricate tapestry of challenges and opportunities inherent in the path toward sustainable and decent employment for all by addressing gender disparities, grappling with the nuances of market economy systems, confronting the realities of the informal sector, and navigating the intricate balance between economic expansion and environmental sustainability.

### 5.1 Gender disparities: breaking the chains of discrimination

The persistence of gender disparities in the labor market is a substantial impediment to achieving SDG 8. Despite tremendous progress in recent years (Fink et al., 2018; Tripathy, 2018; Samuel et al., 2020), women face formidable obstacles, ranging from systemic prejudice to socioeconomic disparities. Gender equality in the workplace is not just a moral issue; it is also a strategic necessity. Research regularly shows that economically empowered women lead to more considerable societal benefits, such as increased economic growth and social cohesion (Costa et al., 2023; Naveen et al., 2023). Comprehensive strategies and targeted activities are required to break down these barriers. Encouragement of female involvement in education and training, promotion of mentorship programs, and advocacy for equal pay are all critical steps toward making the job market a fair playing field for all women (Pignatti, 2016; Tabassum, 2019; Devadas and Kim, 2020).

### 5.2 Market economies paradox: balancing growth and social equity

A closer look into market economic systems shows a paradox at the heart of economic development. While purely market-based labor markets promote economic progress and innovation, they also encourage income inequality, job insecurity, and exploitation (Wray, 2009; Galanis et al., 2017; He, 2022). Because of this dichotomy, economic policy must be complex. Governments and institutions must actively lessen the negative impacts of uncontrolled capitalism by instituting progressive taxation, assuring equitable salaries, and strengthening social safety nets. Furthermore, cultivating a corporate social responsibility culture can incentivize corporations to contribute constructively to society (Verma, 2015; Varona, 2020; Chigbu and Nekhwevha, 2022c), thereby balancing the tensions between economic growth and social equality.

### 5.3 The informal sector: bridging gaps in legal rights and social security

Although the informal sector employs a sizable fraction of the global workforce, it is nevertheless underserved by SDG 8. Workers in informal economies frequently lack legal rights and social security, exposing them to exploitation and poverty (Garzón-Duque et al., 2017; Dwi Winarni et al., 2019). Recognizing the enormous contribution of the informal sector to economies, governments must provide legal protections and social welfare benefits to these people. Formalizing the informal sector through accessible registration processes, tailored financial services, and vocational training can uplift millions, fostering inclusive growth and narrowing disparities (Catherine and Jacob, 2014; Khamis, 2014; Tara et al., 2020).

### 5.4 Balancing economic expansion and environmental sustainability: a delicate act

Economic growth, a crucial aspect of SDG 8, frequently collides with environmental sustainability imperatives. Rapid industrialization and resource-intensive production put a strain on ecosystems (Sharma, 2018; Bandurin and Bandurina, 2021), offering considerable challenges to achieving sustainable development (Kuznetsova et al., 2020). Revolutionary changes in production processes and consumption patterns are required to reach equilibrium. Embracing green technologies, developing circular economies, and investing in renewable energy sources are critical measures (Berg et al., 2018; Adeosun et al., 2023). Simultaneously, raising awareness and cultivating an environmental culture can stimulate behavioral shifts, supporting sustainable practices throughout companies and communities.

As we face these problems head-on, we set the groundwork for a more equitable, sustainable, and prosperous society in which the promises of SDG 8 become lived realities for every individual, regardless of gender, socioeconomic background, or job position.

## 6 Conclusion

This detailed study delves into the underlying complexity of the current labor market by evaluating the complicated web of problems within the goal of Decent Work and Economic Growth – SDG 8. Our aims were designed to elucidate the multifaceted dimensions of this pursuit, and the findings, as presented, highlight the critical need for nuanced and holistic strategies in the face of gender and income inequalities, the inherent injustices of market economies, the often-overlooked realities of informal work, and the imperative of integrating environmental sustainability into economic practices. We discovered that continuing gender gaps in the labor market pose a substantial barrier to realizing SDG 8’s objective. Women continue to encounter enormous obstacles, ranging from discrimination to occupational segregation. Examining market economic systems found a perplexing link between economic development and adequate employment. While capitalism promotes economic growth and innovation, it produces income inequality, insecure work, and exploitation. Our study indicated that the informal sector, which employs a sizable proportion of the global workforce, is still underserved by SDG 8. Workers in this sector lack legal rights and social security and are frequently exploited. Economic expansion often collides with environmental sustainability, creating substantial obstacles to SDG 8. The current emphasis on GDP development, particularly in capitalist cultures, contributes to environmental deterioration, which is aggravated by a lack of decoupling of economic expansion from resource use.

To achieve decent work and economic growth in the 21st century, a paradigm shift is required – one that embraces gender equality, challenges purely market-based labor market conventions, empowers informal workers, and promotes environmental sustainability. Because of the complexities of these difficulties, coordinated efforts, new policies, and a commitment to social justice are required. By confronting these multifarious concerns head-on, nations may pave the way for a future in which decent work is not just a goal but a fundamental human right, providing prosperity for everyone and protecting the well-being of our planet for future generations. This study is a rallying cry, calling governments, scholars, and global citizens to collaborate in breaking down barriers and establishing a path toward a more equal and sustainable society.

### 6.1 Future research direction

Building on the findings and knowledge gaps identified in our study, some potential future research areas can be explored to deepen our understanding of decent work and sustainable economic growth within the context of SDG 8. One of these prospective study directions includes: “Case Studies on Successful Decent Work Initiatives.” Conducting in-depth case studies on countries or regions that have made significant progress in promoting decent work and achieving sustainable economic growth within the framework of SDG 8 can offer valuable insights and best practices for other nations to adopt.

## Author contributions

BC: Conceptualization, Data curation, Formal analysis, Funding acquisition, Investigation, Methodology, Project administration, Resources, Writing – original draft, Writing – review & editing. FN: Conceptualization, Data curation, Formal analysis, Funding acquisition, Investigation, Methodology, Resources, Writing – original draft, Writing – review & editing.

## Footnotes

^1^ unescap.org
